# Ultrastructure of antennal sensilla of four skipper butterflies in *Parnara* sp. and *Pelopidas* sp. (Lepidoptera, Hesperiidae)

**DOI:** 10.3897/zookeys.399.7063

**Published:** 2014-04-08

**Authors:** Yuan Xiangqun, Gao Ke, Yuan Feng, Zhang Yalin

**Affiliations:** 1 Key Laboratory of Plant Protection Resources and Pest Management, Ministry of Education; Entomological Museum, Northwest A&F University, Yangling, Shaanxi 712100, China

**Keywords:** Lepidoptera, Hesperiidae, morphology, fine structure

## Abstract

Most species of *Parnara* and *Pelopidas* (Hesperiidae) are important pests of rice. In this study, the antennal morphology, types of sensilla, and their distribution of four skipper butterflies, including *Parnara guttata* (Bremer & Grey), *Pa. bada* (Moore), *Pelopidas mathias* (Fabricius) and *Pe. agna* (Moore), were observed using a scanning electron microscope. Six distinct morphological types of sensilla were found on the antennae of all of these species: sensilla squamiformia, sensilla trichodea, sensilla chaetica, sensilla auricillica, sensilla coeloconica, and Böhm sensilla. The sensilla trichodea are the most abundant sensilla among the four skipper butterflies, and the sensilla auricillica are confirmed on the antennae of butterflies for the second time. In addition, the possible functions of these sensilla are discussed in the light of previously reported lepidopteran insects, which may provide useful information for further study of the function of these antennal sensilla and for related pests control by applying sex pheromones.

## Introduction

The antennae of insects have various types of sensilla that play important roles in insect behaviors, including host location, feeding, mate attraction and oviposition (Zacharuk 1980; Skiri et al. 2005). Antennal sensilla have been extensively recorded in many insect groups (Amer and Mehlhorn 2006; Sukontason et al. 2004; Bleeker et al. 2004). Although the structure and function of antennal sensillae in Lepidoptera have been well known for decades (Anderson et al. 2000), little research has involved butterflies, especially some important pest species.

*Parnara guttata* (Bremer & Grey), *Parnara bada* (Moore), *Pelopidas mathias* (Fabricius) and *Pelopidas agna* (Moore) are among the most important pests of rice in China. The larvae of these four species feed on the leaves of rice, causing considerable damage and great loss of rice production. So far, the control of rice plant skippers chiefly relies on the use of chemical insecticides, which in turn causes many negative consequences. Biological controls, including the application of sex pheromones, have become increasingly important. Consequently, research of pest antennae has immediate application to the suppression of pests (Smith and Wall 1998). In order to better understand their olfactory system related to the biological control of these four skippers, we researched the type, size, and distribution of antennal sensilla of *Parnara guttata* (Bremer & Grey), *Parnara bada* (Moore), *Pelopidas mathias* (Fabricius) and *Pelopidas agna* (Moore).

## Materials and methods

### Insects

All insects studied are specimens in the entomological museum of Northwest A&F University. More specific information is provided in Table 1.

**Table 1.** Material localities and collection dates.

Species	Collection location	Collection date
*Parnara guttata* (Bremer & Grey)	Huxian County, Shaanxi Province	2009.08.15
	Lantian County, Shaanxi Province	2012.08.15
	Fuzhou City, Fujian Province	2006.07.01
	Zhenkang County, Yunnan Province	2007.07.09
*Parnara bada* (Moore)	Ding’an County, Hainan Province	2002.08.08
	Fuzhou City, Fujian Province	2005.11.19
	Jinghong City, Yunnan Province	2007.07.21
*Pelopidas mathias* (Fabricius)	Hanzhong City, Shaanxi Province	1993.07.23
	Fuzhou City, Fujian Province	2003.12.28
	Minqing County, Fujian Province	2005.10.21
*Pelopidas agna* (Moore)	Wuzhi Mountain, Hainan Province	2007.05.20
	Luxi County, Yunnan Province	2005.08.19

### Scanning electron microscope

The antennae of 10 adults of each of the four species were removed under a microscope (Nikon SMZ1500) by using sharp blades. The antennae were washed for 20 s (four times, each for 5 s) in 70% ethanol solution in an ultrasonic cleaner (KH-250DB; 15°C, 50HZ). After critical point drying, the specimens were attached to a holder using electric adhesive tape, sputter-coated with gold, examined and photographed with a S-4800 SEM (at 10 kV~15 kV).

## Results

### Antennal morphology

**Figure 1.**
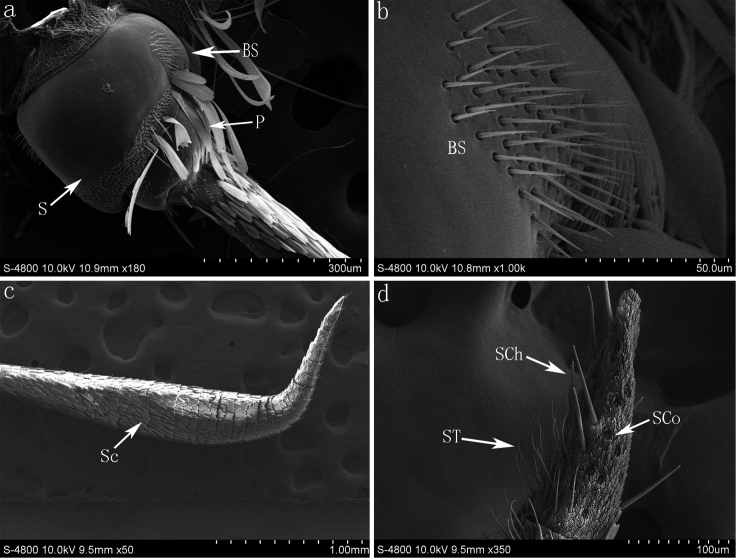
SEM photomicrographs of *Parnara guttata*. (**a**) the scape and pedicel of *Parnara guttata* and location of the Böhm sensilla (**b**) the Böhm sensilla on the scape of antenna (**c**) profile of the flagellum with scales (**d**) profile of last flagellar subsegment of the antenna and the sensilla chaetica, sensilla trichodea and sensilla coeloconica. **S** Scape; **P** Pedicel; **BS** Böhm sensilla; **Sc** Scales; **SCh** sensilla chaetica; **ST** sensilla trichodea; **SCo** sensilla coeloconica.

The antennae of the four studied species of skipper butterflies are located between the compound eyes, and each consists of three components: a basal scape, pedicel, and an elongated flagellum. The first two components consist of a single short segment each one of them (Fig. 1a). The third component, the flagellum, consists of many subsegments. The typical flagellum is thin basally and becomes gradually thicker and curved, covered with scales (Fig. 1c). More types of sensilla are observed on the curved hook (Fig. 1d).

### Types of antennal sensilla

In total, six types of sensilla were observed on the antennae of these four skippers: sensilla squamiformia, sensilla trichodea, sensilla chaetica, sensilla auricillica, sensilla coeloconica, and Böhm sensilla.

#### Sensilla squamiformia (SQ)

**Figure 2.**
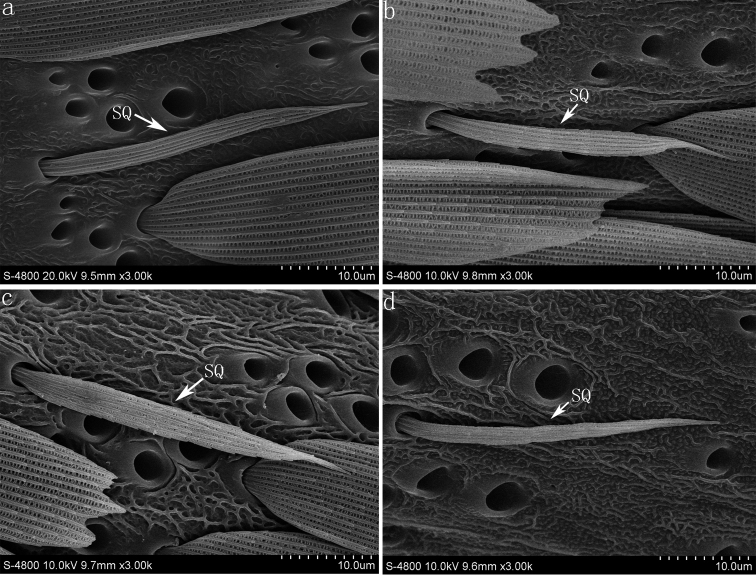
(**a**) sensilla squamiformia on the flagellum of *Parnara guttata* (**b**) sensilla squamiformia of *Parnara bada* (**c**) sensilla squamiformia of *Pelopidas mathias* (**d**) sensilla squamiformia of *Pelopidas agna*. **SQ** sensilla squamiformia.

This type of sensillum is scale-like and elongated with a distal end tapering, found along the base or center flagellum among the scales (Fig. 2a–d). The length of the sensilla squamiformia is 43.5±4.0 μm (*Parnara guttata*), 48.5±6.7 μm (*Parnara bada*), 47.5±5.8 μm (*Pelopidas mathias*), 46.3±3.8 μm (*Pelopidas agna*). The number of sensilla is 1–4 per flagellomere, with the terminal flagellomeres without any among the four skipper butterflies.

#### Sensilla trichodea (ST)

**Figure 3.**
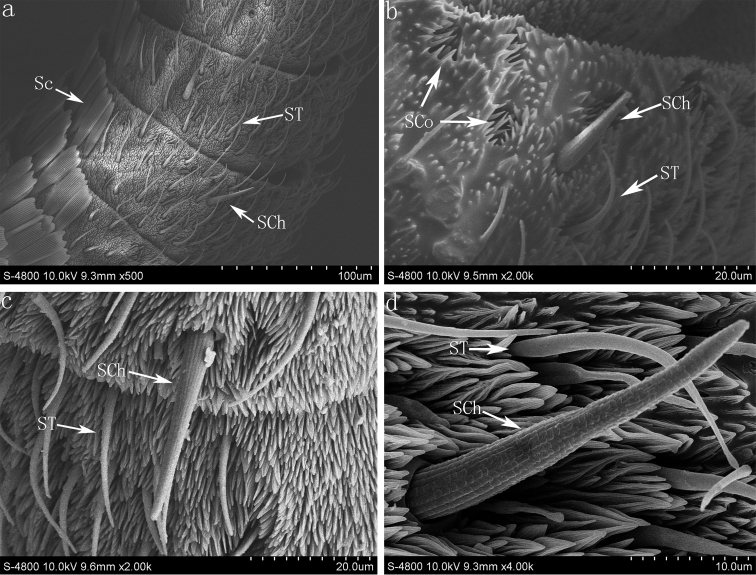
(**a**) The sensilla chaetica, sensilla trichode and scales on the flagellum of *Parnara guttata* (**b**) the sensilla chaetica, sensilla trichode and sensilla coeloconica on the flagellum of *Parnara bada* (**c**) the sensilla chaetica and sensilla trichode on the flagellum of *Pelopidas mathias* (**d**) the sensilla chaetica and sensilla trichode on the flagellum of *Pelopidas agna*. **Sc** Scales; **SCh** sensilla chaetica; **ST** sensilla trichodea; **SCo** sensilla coeloconica.

The sensilla trichodea are hair-like, tapering apically. They occur along the distal segments on the ventral surface (Figs 1d and 3a and b). The surface of the cuticular wall of sensilla trichodea is smooth and the wall pores are not seen with scanning electron microscope (Fig. 3c and d). These sensilla (range 27.1±3.2 μm–28±1.5 μm) are the most abundant with about 32–69 per flagellomere in the four species.

#### Sensilla chaetica (SCh)

The sensilla chaetica have a straight needle-like appearance with a grooved surface (Figs 1d and 3a–c). Each sensilla arise from a round socket, is wide at the base and sharp at the distal end (Fig. 3d). These sensilla (range from 29.5±4.1 μm to 39.5±7.5 μm) are distributed evenly (1–3 per flagellomere) among the scales at the base and center of the flagellomere and among the sensilla trichodea along the flagellum. 4–7 larger sensilla chaetica (80.3±5.8 μm) are distinct and can be found on the terminal segment of flagellum.

#### Sensilla auricillica (SAu)

**Figure 4.**
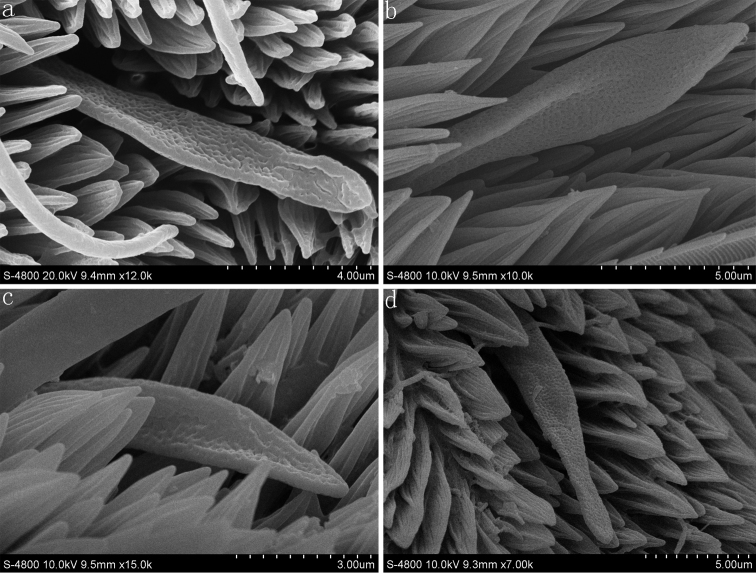
The sensilla auricillica of *Parnara guttata* (**a**) *Parnara bada* (**b**) *Pelopidas mathias* (**c**) and *Pelopidas agna* (**d**).

The sensilla auricillica are short and ear-shaped with a blunt and rounded tip. The surface of the cuticular wall of ear-shaped sensilla is covered with small pores (Fig. 4a–d). These sensilla are only scattered along the distal end of the flagellum. These sensilla (about 6–14 per flagellomere) are very similar and the length varies from 12.8±3.4 μm to 15.5±0.3 μm among all four skipper butterflies.

#### Sensilla coeloconica (SCo)

**Figure 5.**
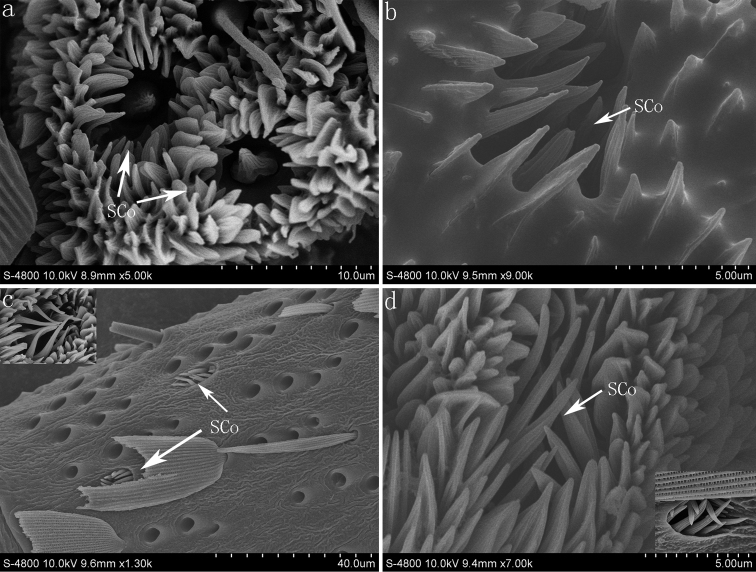
The sensilla coeloconica of *Parnara guttata* (**a**) *Parnara bada* (**b**) *Pelopidas mathias* (**c**) and *Pelopidas agna* (**d**). **SCo** sensilla coeloconica

The sensilla coeloconica consist of a submerged central peg with a grooved surface and blunt tip surrounded by a ring of cuticular spines (Figs 3b, 5a–d). They are found on the distal end of the flagellum (about 6–12 per flagellomere) in the four species (Fig. 1d). In *Pelopidas mathias* and *Pelopidas agna*, these sensilla are also found occasionally on the base or center of the flagellomere as they are difficult to discern since the scales will conceal them (Fig. 5c and d).

#### Böhm sensilla (BS)

Böhm sensilla are spine-like structures with smooth cuticles. Böhm sensilla, in clusters, are inserted to the base of scape and pedicel segments only (Fig. 1a and b). Each cluster has approximately 56, 59, 34 and 32 sensilla respectively among *Parnara guttata*, *Parnara bada*, *Pelopidas mathias* and *Pelopidas agna*.

### Discussion

Sensilla squamiformia are commonly present in lepidopteran insects (Faucheux 1999). The sensilla squamiformia found in this study are similar in shape and distribution to those reported in two other butterfly species, *Teinopalpus aureus* Mell and *Heliophorus phoenicoparyphus* (Holland) (Jiang et al. 2000; Xu and Wang 2013); but the shape and distribution of these sensilla are different from several reported moth species, *Synanthedon scitula* (Harris), *Coleophora* sp. and *Zamagiria dixolophella* Dyar (Frank et al. 2010; Faucheux 2011; Gómez et al. 2003) and also different from Coleoptera (Hix et al. 2003; Gao et al. 2013). These aporous sensilla are inferred to have a mechanoreceptive function (Schneider 1964).

We identified only one type of sensilla trichodea among the four skipper butterflies. However, studies of other moth species have shown those sensilla can be divided into more subtypes according to their size and pore density (Faucheux 1999). For example, three subtypes of these sensilla are found on *Synanthedon scitula* and *Ostrinia nubilalis* (Hübner) (Frank et al. 2010; Hallberg et al. 1994). Furthermore, the number of these sensilla in Bombycidae, Tortricidae, Tineidae and Pyralidae is significantly different between male and female antennae (Steinbrecht 1973; George and Nagy 1984; Faucheux 1987; Wang et al. 2008). The accumulated studies have shown that multiporous sensilla trichodea are associated with olfactory reception of sex pheromones (Hansson et al. 1995; Ebbinghaus et al. 1997; Ma and Du 2000).

Sensilla chaetica found in this study are similar in structure to those reported for the Lycaenidae: *Chilades pandava* (Horsfield) and *Heliophorus phoenicoparyphus* (Jian et al. 2011; Xu and Wang 2013). These sensilla have also been observed in many other moth species, viz, *Cydia nigricana* (Fabricius), *Bactra furfurana* (Haworth), and *Zamagiria dixolophella* (Wall 1978; Razowski and Wojtusiak 2004; Gómez et al. 2003). Several studies noted that these uniporous sensilla to be contact chemoreceptors (Altner and Prillinger 1980; Hallberg et al. 1994).

Although the sensilla auricillica have been easily observed in the months, these sensilla on the antenna of butterfly was described for the first time in *Pieris rapae* L. (Faucheux 1996, 1999). Our observations on the Hesperiidae confirm their presence in the butterflies. Several studies on moth species considered multiporous sensilla auricillica as olfactory receptors for plant volatiles (Boekh et al. 1965; Kaissling 1971). Others suggest they respond to sex pheromone compounds (Ebbinghaus et al. 1997; Anderson et al. 2000; Faucheux 2006).

In this study, the multiporous sensilla coeloconica closely resemble those observed in many other Lepidoptera. This type of sensilla is considered to have a humidity and temperature sensitive function (Altner et al. 1977). Pophof (1997) reported that in *Bombyx mori* L., they are sensitive to plant volatiles and are possibly involved in the selection of oviposition sites. Sensilla coeloconica were found under the scales on the antennae of *Pelopidas mathias* and *Pelopidas agna*, as has not been reported in other insects.

Böhm sensilla observed here are morphologically similar to those presented in other families of Lepidoptera, e.g., Pyralidae, Tortricidae, Sesiidae (Gómez et al. 2003; Gómez and Carrasco 2008; Frank et al. 2010). The absence of dendrite in the sensillum lumen and the presence of a tubular body at the base of the hair, observed in the Böhm sensilla of *Tineola bisselliella* Humm. (Faucheux 1987) are characteristic of the mechanoreceptors with a proprioceptive function (Schneider 1964; Faucheux 1999).

In summary, we identified six different types of sensilla on the antennae of *Parnara guttata*, *Parnara bada*, *Pelopidas mathias* and *Pelopidas agna*. The external morphology and distribution of these sensilla among *Parnara* and *Pelopidas*, is very similar and also somewhat similar to other reported Lepidoptera. However, documents on morphology of antennal sensilla in butterfly species are still very limited yet. Further exploration on antennal sensilla of these group need merits to be conducted, which may provide useful information for taxonomy and phylogeny of Lepidoptera, and for further studies on the function of antennal sensilla and related pests control by application of sex pheromones.
